# Prevalence of sleep disturbance and associated factors among nurses in Chinese tertiary public hospitals: a national cross-sectional study

**DOI:** 10.3389/fpubh.2025.1735543

**Published:** 2026-01-21

**Authors:** Chunhui Bin, Xuting Li, Yusheng Tian, Zengyu Chen, Yiting Liu, Meng Ning, Jiaxin Yang, Qiang Yu, Chongmei Huang, Dan Zhang, Jianghao Yuan, Zhenhui Ren, Yamin Li

**Affiliations:** 1 Xiangya Nursing School of Central South University, Changsha, Hunan, China; 2 Clinical Nursing Teaching and Research Section, The Second Xiangya Hospital, Central South University, Changsha, Hunan, China; 3 Department of Thoracic Surgery, The Second Xiangya Hospital of Central South University, Changsha, Hunan, China; 4 National Clinical Research Center for Mental Disorders, The Second Xiangya Hospital of Central South University, Changsha, Hunan, China; 5 Department of Psychiatry, National Clinical Research Center for Mental Disorders, The Second Xiangya Hospital of Central South University, Changsha, Hunan, China; 6 School of Nursing, University of Washington, Seattle, WA, United States; 7 Department of Emergency, The Second Xiangya Hospital of Central South University, Changsha, Hunan, China; 8 School of Computer Science and Engineering, Central South University, Changsha, Hunan, China; 9 School of Nursing at Ningxia Medical University, Yinchuan, Ningxia, China; 10 Hunan Provincial People's Hospital (The First Affiliated Hospital of Hunan Normal University), Changsha, Hunan, China

**Keywords:** cross-sectional study, nurse, occupational health, sleep disturbance, workplace factors

## Abstract

**Background:**

Sleep disturbance is a critical occupational health issue among nurses, jeopardizing their well-being and patient safety. Identifying its prevalence and modifiable factors is essential for developing effective interventions.

**Objectives:**

This study aimed to determine the national prevalence of sleep disturbance and explore associated demographic, occupational, and behavioral factors among Chinese nurses.

**Methods:**

In this nationwide cross-sectional study, we analyzed baseline data from the Nurses’ Mental Health Study (NMHS), a primary database established and maintained by the authors. A total of 132,910 nurses from 67 tertiary public hospitals in China were included between December 2023 and January 2024. Data were collected electronically via a structured questionnaire. Chi-square tests and binary logistic regression were used to identify factors associated with sleep disturbances.

**Results:**

The overall prevalence of sleep disturbances was 24.1%. Difficulty initiating sleep (13.3%), difficulty maintaining sleep (15.5%), and early morning awakening (15.4%) were most common, with higher rates in critical care, emergency, pediatrics, and obstetrics. Factors associated with increased odds of sleep disturbance included age (30–39 years), female sex, intermediate professional title, lower education, longer working experience, night shift work, smoking, alcohol use, and physical inactivity.

**Conclusion:**

Sleep disturbance is prevalent among Chinese nurses and associated with scheduling patterns and health behaviors. To mitigate risk, healthcare organizations should optimize shift schedules, enhance support for high-risk nurses, and allocate resources to high-burden clinical departments. Nurses are also encouraged to adopt healthier lifestyles to improve sleep quality and occupational safety.

**Clinical trial registration:**

Identifier ChiCTR2300072142.

## 1 Background

Adequate sleep, beyond a physiological necessity, is a non-negotiable prerequisite for nursing competence and patient safety (1, 2). Sustained vigilance and emotional stability are essential for delivering high-quality care, yet these capacities are compromised by the high global prevalence of sleep disturbances among nurses (3).

Insomnia, the most common type of sleep disturbance, has serious implications for health and work performance (4). It is clinically defined by the DSM-IV as persistent difficulties with sleep initiation, duration, consolidation, or quality—despite adequate opportunity for sleep—resulting in daytime impairment, and is sub-classified into difficulty initiating sleep (DIS), difficulty maintaining sleep (DMS), and early morning awakening (EMA) (5). However, a significant methodological discrepancy exists: most studies on nurses’ sleep rely on self-report instruments (e.g., PSQI, ISI) rather than clinical diagnoses (1, 2). Consequently, we employ the broader term “sleep disturbance” to encompass all sleep-related complaints assessed in this study, which may not meet the full DSM-IV criteria for insomnia.

Nurses are at particularly high risk for sleep disturbance, primarily due to circadian rhythm disruption from shift work (3, 6). The consequences extend beyond impaired personal well-being to include reduced immunity, increased emotional distress, and higher morbidity (7). Crucially, sleep-related cognitive impairment elevates the likelihood of clinical errors, thereby jeopardizing patient safety (8, 9).

Despite these findings, evidence on the prevalence and risk factors lacks national representativeness and is subject to significant methodological limitations (3, 10). Although global and regional meta-analyses report high pooled prevalence rates [e.g., 34.8–61.0% globally (3, 11); 39.2–49.9% in China (12, 13)], these estimates are derived from studies often limited by single-center designs, small sample sizes, and convenience sampling. Research on risk factors is similarly constrained, as variable selection is often selective or data-driven rather than being guided by a comprehensive theoretical framework (10, 14). This narrow methodology introduces selection bias, severely limits generalizability, and potentially inflates prevalence estimates. A critical and unaddressed gap is whether risk factors differ across sleep disturbance sub-types (DIS, DMS, EMA)—an issue our study aims to address.

To overcome these gaps, a nationwide study targeting the nursing workforce within Chinese tertiary hospitals is essential. Within China’s unique hierarchical medical system, tertiary hospitals bear the core responsibility of treating severe cases (15, 16). Their nurses face higher workloads, more frequent shift work, and complex patient care demands, making their sleep disturbance patterns more typical of the professional group and helping to avoid the bias of single-institution samples (17, 18).

To analyze this complex interplay of factors, a comprehensive theoretical framework is required. We therefore adopted Krieger’s Eco-social Model, which conceptualizes health outcomes as emerging from the causal pathway of “social structure shaping environmental exposures, which in turn affect individual health” (19). This framework is uniquely suited to analyzing nurses’ sleep, as their sleep health is shaped by factors operating across macro, mezzo, and micro levels.

At the macro-level, organizational systems, such as the requirement for 24/7 shift work, directly disrupt circadian rhythms (20); at the mezzo-level, workplace environments characterized by high nurse-to-patient ratios and exposure to workplace violence increase psychological stress, indirectly impairing sleep (20, 21); at the micro-level, a constellation of individual factors is critical, including psycho-social and demographic traits (e.g., low social support, age, sex, education) (22–24), modifiable lifestyle behaviors and comorbidities (e.g., caffeine use, physical inactivity, chronic pain, and depression or anxiety) (23–26), and work–family conflict (27), which adds a significant layer of emotional strain.

This national, multi-center study aims to overcome previous limitations by recruiting a large, geographically dispersed sample from multiple tertiary hospitals. Our objectives are to: (1) determine the nationally representative prevalence of sleep disturbance and its sub-types (DIS, DMS, EMA); (2) identify key generalizable risk factors across demographic, occupational, and lifestyle domains; and (3) critically examine whether these risk factors operate differentially across the three insomnia sub-types. The results will provide a robust evidence base for developing targeted interventions and offer globally applicable insights for enhancing nurse well-being and patient safety in high-intensity healthcare settings.

## 2 Methods

### 2.1 Study design and participants

This cross-sectional study utilized primary data from the Nurses’ Mental Health Study (NMHS), a nationwide cohort initiated and conducted by the authors across 67 tertiary public hospitals in 31 Chinese provinces between December 2023 and January 2024. Ethical approval (LYF20230048) was obtained from the Institutional Review Board of the Second Xiangya Hospital, Central South University prior to initiation.

Using a two-stage cluster sampling approach, we first randomly selected 2–3 tertiary public hospitals per province from 31 Chinese administrative regions. All hospitals met predefined criteria: (1) general tertiary classification; (2) ≥ 2,000 beds or provincial top-5 ranking by bed capacity; (3) multidisciplinary service capacity [as prespecified in protocol (28)]. All full-time registered nurses (≥18 years) were eligible, excluding those on long-term leave, retired nurses, or interns.

### 2.2 Sample size determination

The minimum sample size was 38,416 based on detecting events with 1% prevalence at ±0.1% absolute precision (two-sided *α* = 0.05). We recruited all eligible nurses at selected hospitals to ensure complete workforce coverage, issuing 147,832 invitations. This comprehensive approach enhanced representativeness and statistical power for subgroup analyses.

### 2.3 Data collection

**Figure 1:**
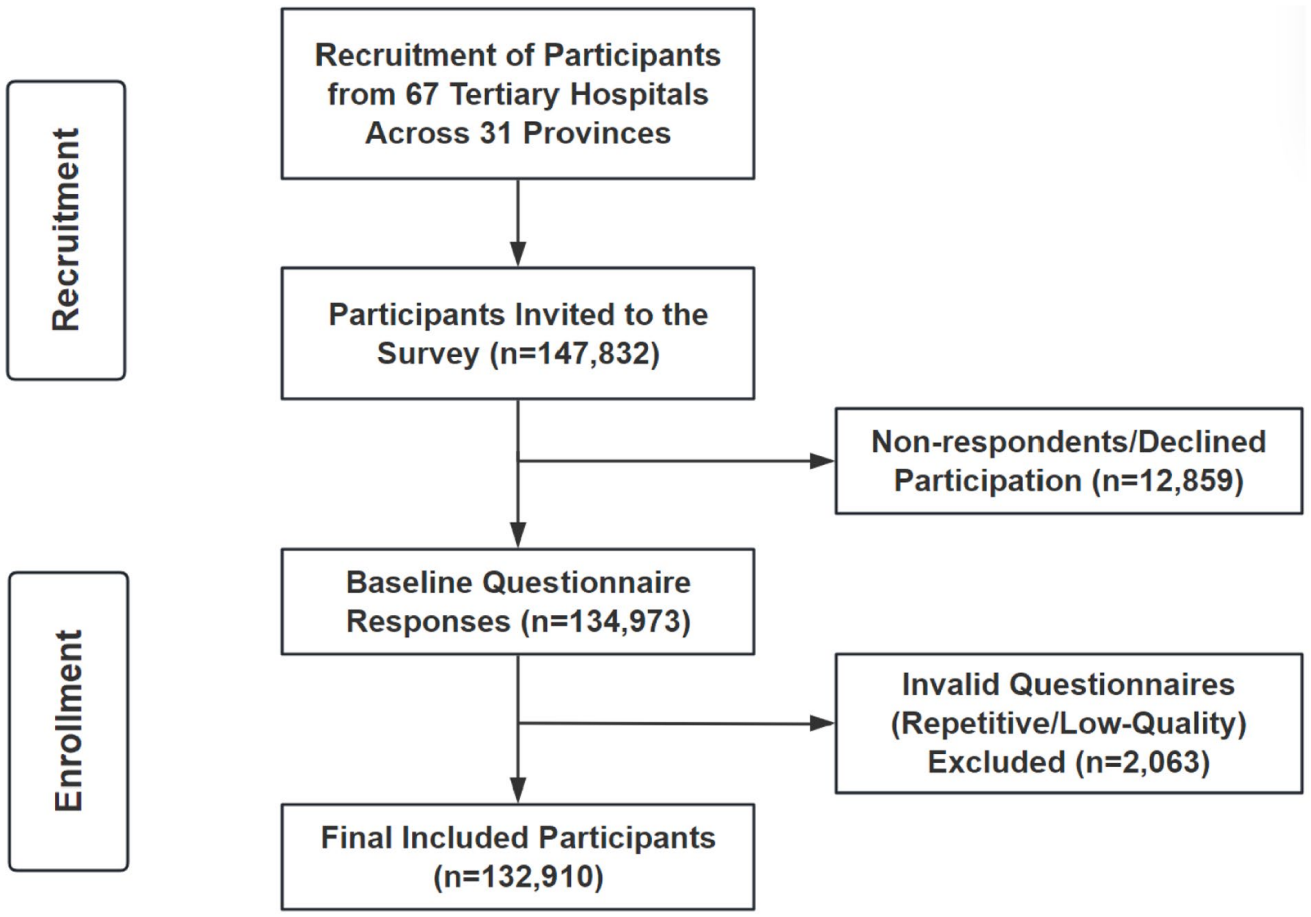
Flowchart of the study participants. Invalid questionnaires (*n* = 2063) were excluded for duplicate responses or ≥3 logical inconsistencies (e.g., conflicting age/working years).

Trained research assistants administered questionnaires electronically via the Wenjuanxing® platform,^1^ a validated web-based survey platform widely implemented in Chinese clinical research (29, 30). Participants self-completed questionnaires (average duration: 15 min) with real-time logic checks to prevent invalid entries. From 147,832 invitations, 134,973 responses were received (91.3% response rate); 2,063 responses were excluded due to duplicate entries or ≥3 logical inconsistencies (e.g., conflicting age/working years), leaving 132,910 valid questionnaires for analysis (Figure 1).

### 2.4 Recruitment

The Chinese Nursing Association coordinated hospital recruitment. Site nursing directors appointed liaisons to distribute encrypted access codes. Prior to participation, all nurses reviewed electronic informed consent documents detailing study objectives, risks, benefits, and confidentiality measures, then actively selected “Agree” or “Disagree” to indicate voluntary participation.

### 2.5 Measurements

NMHS has developed a baseline questionnaire (see Additional file 1), which was derived from standardized questionnaires and scales.

#### 2.5.1 Demographic and occupational variables

Basic demographic characteristics were gathered through standardized questions, which included age, sex, working experience, job title, education level, and marital status. Department and night shift status were determined through standard yes/no questions. The definition of night work referenced the legal guidelines of each country. For this study, night work was defined as working for a minimum of 3 h between 12:00 a.m. and 6:00 a.m.

#### 2.5.2 Lifestyle variables

Participants’ smoking status was assessed using a questionnaire designed based on Li-Ming Li′s publication, “Large Population Cohort Studies - Appropriate Techniques for Surveys,” specifically Chapter 3, Section 2. In addition, “non-smoking” was included as a distinct response option.

Drinking frequency was evaluated through the Alcohol Use Disorder Identification Test (AUDIT) developed by the World Health Organization (WHO), which asks, “In the past year, how often do you drink alcohol?”

Exercise frequency was measured with a question from the Medscape Physician Lifestyle & Happiness Report 2022: “How often do nurses exercise?”

Information on napping was gathered through standard yes/no questions. While naptime was categorized into no napping (0 min), 1–30 min, 31–60 min, 61–90 min, and over 90 min, as measured by the Insomnia Severity Index (ISI).

#### 2.5.3 Sleep disturbances

Information of three basic types of sleep disturbance symptoms occurring in the past month was ascertained by asking three standard questions (31): (1) DIS “Did you ever have difficulties in falling asleep?’; (2) DMS: “Did you ever have difficulties in maintaining sleep?”; (3) EMA: “Did you ever wake up in the middle of the night or early morning and have difficulties in falling asleep again?.” Respondents could choose from three options: “No,” “Sometimes” or “Often.” Patients who answered “Often” to any of these questions were classified as having “sleep disturbance.” This classification aligns with epidemiological studies capturing clinically significant symptoms (31).

#### 2.5.4 Perceived stress

Perceived stress was measured using the 4-item Perceived Stress Scale (PSS-4) (32). This scale assesses the degree to which situations in one’s life in the last month are appraised as stressful, based on an individual’s self-report of feelings and thoughts. The PSS-4 consists of four items rated on a 5-point Likert scale, ranging from 0 (Never) to 4 (Very Often). The total score ranges from 0 to 16. Specifically, items 2 and 3 are phrased positively (e.g., feeling confident about handling personal problems) and are reverse-scored during calculation. Higher total scores indicate a higher level of perceived stress.

#### 2.5.5 Data analysis

Data analysis was performed using SPSS (version 27.0; IBM Corp.) and R software (version 4.5.1). Chi-square tests were used to compare demographic characteristics between nurses with and without sleep disturbance. Continuous variables, such as PSS-4 scores, were presented as mean ± standard deviation, and independent samples *t*-tests were used for group comparisons. Missing values in independent variables (6.2%) were handled via multiple imputation by chained equations (MICE), creating 5 imputed datasets. Multicollinearity was assessed by variance inflation factors (VIF) and tolerance (VIF < 10; tolerance > 0.1), and no severe multicollinearity was detected. Multi-variable logistic regression (Forward: Likelihood Ratio method) was applied to identify predictors of sleep disturbance subtypes; only variables significantly contributing to the model were retained, adjusted odds ratios (ORs) with 95% confidence intervals (CIs) were derived from pooled results across the imputed datasets. To capture the potential non-linear association between age and sleep disturbance, we employed a restricted cubic spline (RCS) with 5 knots within the logistic regression framework (using the rms package in R) and visualized the predicted probabilities. Statistical significance was set at a two-tailed *p* < 0.05. A detailed description of the methods is available in the Supplementary material.

## 3 Results

A total of 147,832 registered nurses were recruited and 132,910 high-quality questionnaires were returned with a valid response rate of 89.9%. Supplementary Table S1 shows the total number of nurses in the 67 tertiary public hospitals and the percentage of the sample represented by the 67 hospitals.

### 3.1 Prevalence of sleep disturbances among nurses in China

Table 1 presents the seven mutually exclusive patterns of sleep disturbance. Strikingly, the simultaneous presence of all three symptoms (DIS + DMS + EMA) emerged as the most prevalent pattern (7.2%), surpassing any single isolated subtype (range: 3.2% ~ 4.5%). This finding underscores that sleep disturbance in this population is predominantly characterized by complex comorbidities rather than solitary symptoms.

**Table 1:** Prevalence of seven mutually exclusive patterns of sleep disturbance.

Group	*N*	%
No sleep disturbance	100,933	75.9
Sleep disturbance	31,977	24.1
Isolated subtypes		
Only DIS (difficulty initiating sleep)	4,290	3.2
Only DMS (difficulty maintaining sleep)	4,533	3.4
Only EMA (early morning awakening)	5,992	4.5
Comorbid subtypes		
DIS + DMS	2,709	2.0
DIS + EMA	1,090	0.8
DMS + EMA	3,731	2.8
Combined type		
DIS + DMS + EMA	9,632	7.2
Total	132,910	100

### 3.2 Characteristics of nurses with sleep disturbances

Table 2 shows a comparison of demographic characteristics between participants with and without sleep disturbance (DIS, DMS, and EMA). Individuals experiencing any of these symptoms are categorized as having sleep disturbances. Most nurses were female (93.5%), aged 30–39 (50.0%), married (68.9%), holding primary titles (51.6%), possessed bachelor’s degrees (87.1%), working night shifts (72.2%), had more than 10 years of working experience (53.7%), not taking naps (43.1%), had never smoked (96.3%) and drank (61.2%) and engaged in regular exercise (58.7%). However, each group also had a notable percentage of intermediate titles, secondary or higher vocational education, former smokers, and individuals who did not exercise. Those with sleep disturbances were more likely to be females, older, have more than 10 years of working experience, possess a lower education level, hold senior job titles, work night shifts, not take naps, smoke, drink alcohol, and report no regular exercise. Significant differences were also observed between the two groups in terms of marital status.

**Table 2:** Demographic and clinical characteristics of the study sample (*n* = 132910).

Variables	Sleep disturbance				DIS				DMS				EMA			
	No (*n* = 100933)		Yes (*n* = 31977)		No (*n* = 115189)		Yes (*n* = 17721)		No (*n* = 112305)		Yes (*n* = 20605)		No (*n* = 112465)		Yes (*n* = 20445)	
	*n*	%	*n*	%	*n*	%	*n*	%	*n*	%	*n*	%	*n*	%	*n*	%
Demographic variables																
Age group(yr)*																
<30	34032	81.2	7885	18.8	37232	88.8	4685	11.2	37448	89.3	4469	10.7	37806	90.2	4111	9.8
30–39	49111	74.0	17292	26.0	57222	86.2	9181	13.8	54980	82.8	11423	17.2	55168	83.1	11235	16.9
≥40	17776	72.3	6795	27.7	20720	84.3	3851	15.7	19862	80.8	4709	19.2	19475	79.3	5096	20.7
*p* value	<0.001				<0.001				<0.001				<0.001			
*χ^2^*	948.548				300.025				1148.426				1663.217			
Sex																
Male	6753	77.9	1913	22.1	7560	87.2	1106	12.8	7587	87.5	1079	12.5	7397	85.4	1269	14.6
Female	94180	75.8	30064	24.2	107629	86.6	16615	13.4	104718	84.3	19526	15.7	105068	84.6	19176	15.4
*p* value	<0.001				0.106				<0.001				0.049			
*χ^2^*	19.98				2.612				65.92				3.891			
Education level																
Secondary or higher vocational education	8705	74.5	2972	25.5	9868	84.5	1809	15.5	9718	83.2	1959	16.8	9697	83.0	1980	17.0
Undergraduate	87655	75.7	28121	24.3	100306	86.6	15470	13.4	97670	84.4	18106	15.6	97847	84.5	17929	15.5
Bachelor's degree or above	4573	83.8	884	16.2	5015	91.9	442	8.1	4917	90.1	540	9.9	4921	90.2	536	9.8
*p* value	<0.001				<0.001				<0.001				<0.001			
*χ^2^*	200.256				176.525				147.076				152.789			
Marital status																
Never married	30156	79.4	7836	20.6	33006	86.9	4986	13.1	33602	88.4	4390	11.6	33809	89.0	4183	11.0
Married	68609	74.9	23008	25.1	79603	86.9	12014	13.1	76190	83.2	15427	16.8	76141	83.1	15476	16.9
Separated, widowed or divorced	2168	65.7	1133	34.3	2580	78.2	721	21.8	2513	76.1	788	23.9	2515	76.2	786	23.8
*p* value	<0.001				<0.001				<0.001				<0.001			
*χ^2^*	491.219				212.092				753.272				898.534			
Occupational variables																
Working experience (yr)**																
<5	22520	83.2	4551	16.8	24375	90.0	2696	10.0	24597	90.9	2474	9.1	24768	91.5	2303	8.5
5–9	24792	77.1	7368	22.9	28048	87.2	4112	12.8	27590	85.8	4570	14.2	27914	86.8	4246	13.2
≥10	51900	72.7	19481	27.3	60827	85.2	10554	14.8	58220	81.6	13161	18.4	57883	81.1	13498	18.9
*p* value	<0.001				<0.001				<0.001				<0.001			
*χ^2^*	1211.148				415.757				1353.67				1789.404			
Job title^a^																
Primary	53636	78.2	14957	21.8	59987	87.5	8606	12.5	59402	86.6	9191	13.4	59781	87.2	8812	12.8
Intermediate	42383	73.1	15569	26.9	49607	85.6	8345	14.4	47498	82.0	10454	18.0	47383	81.8	10569	18.2
High	4914	77.2	1451	22.8	5595	87.9	770	12.1	5405	84.9	960	15.1	5301	83.3	1064	16.7
*p* value	<0.001				<0.001				<0.001				<0.001			
*χ^2^*	443.015				102.212				517.122				710.442			
Nightshift																
No	28556	77.2	8419	22.8	32726	88.5	4249	11.5	31358	84.8	5617	15.2	31153	84.3	5822	15.7
Yes	72377	75.4	23558	24.6	82463	86.0	13472	14.0	80947	84.4	14988	15.6	81312	84.8	14623	15.2
*p* value	<0.001				<0.001				0.051				0.023			
*χ^2^*	78.968				173.918				31.846				24.667			
Lifestyle variables																
Nap time(min)																
No	41848	73.0	15499	27.0	48330	84.3	9017	15.7	47272	82.4	10075	17.6	47452	82.7	9895	17.3
<30	15061	73.0	5577	27.0	17566	85.1	3072	14.9	16803	81.4	3835	18.6	16751	81.2	3887	18.8
30–60	36398	80.1	9025	19.9	40766	89.7	4657	10.3	39866	87.8	5557	12.2	39799	87.6	5624	12.4
>60	7626	80.3	1876	19.7	8527	89.7	975	10.3	8364	88.0	1138	12.0	8463	89.1	1039	10.9
*p* value	<0.001				<0.001				<0.001				<0.001			
*χ^2^*	917.631				793.057				804.405				806.856			
Smoke																
Never	97602	76.3	30346	23.7	111314	87.0	16634	13.0	108397	84.7	19551	15.3	108553	84.8	19395	15.2
Currently	3018	68.1	1412	31.9	3489	78.8	941	21.2	3540	79.9	890	20.1	3523	79.5	907	20.5
Used to	313	58.8	219	41.2	386	72.6	146	27.4	368	69.2	164	30.8	389	73.1	143	26.9
*p* value	<0.001				<0.001				<0.001				<0.001			
*χ^2^*	242.801				350.518				171.372				147.591			
Drink																
Never	63584	78.2	17711	21.8	71743	88.3	9552	11.7	69731	85.8	11564	14.2	69703	85.7	11592	14.3
<1/month	32645	73.5	11783	26.5	37843	85.2	6585	14.8	37030	83.3	7398	16.7	37200	83.7	7228	16.3
2–4/month	3807	66.9	1884	33.1	4525	79.5	1166	20.5	4461	78.4	1230	21.6	4494	79.0	1197	21.0
2–3/week	589	62.5	353	37.5	699	74.2	243	25.8	712	75.6	230	24.4	704	74.7	238	25.3
>4/week	308	55.6	246	44.4	379	68.4	175	31.6	371	67.0	183	33.0	364	65.7	190	34.3
*p* value	<0.001				<0.001				<0.001				<0.001			
*χ^2^*	850.495				800.12				495.425				468.18			
Exercise																
Never	40091	73.0	14837	27.0	46593	84.8	8335	15.2	45102	82.1	9826	17.9	45451	82.7	9477	17.3
Currently	60842	78.0	17140	22.0	68596	88.0	9386	12.0	67203	86.2	10779	13.8	67104	86.1	10968	14.1
*p* value	<0.001				<0.001				<0.001				<0.001			
*χ^2^*	465.728				285.641				420.454				325.868			
PSS-4	1.12±0.92		1.64±0.94		1.17±0.93		1.72±0.96		1.16±0.93		1.70±0.95		1.17±0.93		1.67±0.97	
*p* value	<0.001				<0.001				<0.001				<0.001			
*t’*	−85.668				−71.029				−75.796				−68.677			

DIS, difficulty initiating sleep; DMS, difficulty maintaining sleep; EMA, early morning awakening. PSS-4, Perceived Stress Scale 4. *19 invalid data in the age group. **2298 invalid data in the working experience group.

a China’s professional title system for nurses is hierarchical, consisting of three levels and five titles, with progressively increasing requirements for academic degree and working experience at each level. Primary title includes Nurse and Nurse Practitioner; Intermediate title includes Supervisor Nurse (Nurse in charge); High title includes Associate Chief Nurse and Chief Nurse.

Supplementary Table S2 displays the prevalence of sleep disturbances categorized by department. Notably, departments such as Critical Care (ICU) (25.9%, SE = 0.4), Emergency (26.5%, SE = 0.5), Pediatrics (25.7%, SE = 0.7), and Obstetrics (26.4%, SE = 0.5) exhibited relatively high prevalence rates of sleep disturbances. In contrast, psychiatry (17.8%, SE = 1.3) generally reported a lower prevalence compared to other departments.

### 3.3 Influential factors of sleep disturbances among nurses

The results of the multi-variable logistic regression analyses (Table 3) showed associations between variables and sub-types of sleep disturbances among nurses after adjusting for age, sex, education and marital status.

**Table 3:** Factors associated with sleep disturbance sub-types among Chinese nurses: a multi-variable adjusted logistic regression analysis.

Predictor	Sleep disturbance			DIS			DMS			EMA		
	95%CI^a^	aOR	*p*	95%CI	aOR	*p*	95%CI	aOR	*p*	95%CI	aOR	*p*
Demographic variables												
Age group(yr)												
<30	Ref.			Ref.			Ref.					
30–39	1.167–1.288	1.226	<0.001***	1.137–1.256	1.195	<0.001***	1.255–1.418	1.334	<0.001***	1.176–1.325	1.248	<0.001***
≥40	1.339–1.520	1.427	<0.001***	1.362–1.540	1.448	<0.001***	1.713–1.984	1.844	<0.001***	1.435–1.659	1.543	<0.001***
Female	1.316–1.489	1.400	<0.001***	1.271–1.441	1.353	<0.001***	1.002–1.160	1.078	0.043*	1.397–1.631	1.510	<0.001***
Education level												
Secondary or higher vocational education	Ref.			Ref.			Ref.			Ref.		
Undergraduate	0.850–0.932	0.890	<0.001***	0.856–0.940	0.897	<0.001***	0.817–0.911	0.863	<0.001***	0.826–0.921	0.872	<0.001***
Bachelor's degree or above	0.524–0.623	0.571	<0.001***	0.501–0.596	0.546	<0.001***	0.455–0.561	0.505	<0.001***	0.468–0.577	0.519	<0.001***
Marital status												
Never married	Ref.			Ref.			Ref.			Ref.		
Married	0.905–0.980	0.942	<0.001***	0.936–1.016	0.975	<0.001***	1.049–1.158	1.102	<0.001***	1.086–1.198	1.141	<0.001***
Separated, widowed or divorced	1.174–1.386	1.276	<0.001***	1.108–1.316	1.208	<0.001***	1.175–1.427	1.295	<0.001***	1.241–1.507	1.368	<0.001***
Occupational variables												
Working experience (yr)												
<5	Ref.			Ref.			Ref.			Ref.		
5–9	1.272–1.399	1.334	<0.001***	1.260–1.389	1.323	<0.001***	1.199–1.355	1.275	<0.001***	1.221–1.376	1.296	<0.001***
≥10	1.422–1.596	1.507	<0.001***	1.416–1.592	1.502	<0.001***	1.457–1.681	1.565	<0.001***	1.378–1.585	1.478	<0.001***
Job title^b^												
Primary	Ref.					0.607			0.755			0.434
Intermediate	1.017–1.087	1.051	0.003**	–			–			–		
High	0.802–0.928	0.863	<0.001***	–			–			–		
Nightshift	1.192–1.271	1.231	<0.001***	1.151–1.229	1.189	<0.001***	1.097–1.183	1.139	<0.001***	1.112–1.200	1.155	<0.001***
Pressure (PSS-4)	1.252–1.267	1.259	<0.001***	1.252–1.267	1.259	<0.001***	1.253–1.269	1.261	<0.001***	1.270–1.287	1.279	<0.001***
Lifestyle variables												
Nap time(min)												
No nap	Ref.			Ref.			Ref.			Ref.		
<30	0.935–1.007	0.970	0.113	0.945–1.021	0.982	0.358	0.994–1.085	1.038	0.094	0.981–1.070	1.024	0.280
30–60	0.641–0.681	0.661	<0.001***	0.679–0.723	0.701	<0.001***	0.672–0.723	0.697	<0.001***	0.654–0.704	0.678	<0.001***
>60	0.627–0.699	0.662	<0.001***	0.659–0.737	0.697	<0.001***	0.581–0.668	0.623	<0.001***	0.629–0.720	0.673	<0.001***
Smoke												
Never	Ref.			Ref.			Ref.			Ref.		
Currently	1.226–1.431	1.325	<0.001***	1.226–1.431	1.325	<0.001***	1.128–1.347	1.232	<0.001***	1.218–1.458	1.333	<0.001***
Used to	1.543–2.243	1.860	<0.001***	1.543–2.243	1.860	<0.001***	1.336–2.020	1.643	<0.001***	1.755–2.621	2.144	<0.001***
Drink												
Never	Ref.			Ref.			Ref.					
<1/month	1.273–1.345	1.308	<0.001***	1.238–1.310	1.273	<0.001***	1.100–1.176	1.137	<0.001***	1.153–1.232	1.192	<0.001***
2–4/month	1.650–1.863	1.753	<0.001***	1.547–1.754	1.647	<0.001***	1.344–1.552	1.445	<0.001***	1.449–1.672	1.557	<0.001***
>=2/week	1.977–2.456	2.204	<0.001***	1.769–2.218	1.980	<0.001***	1.678–2.145	1.897	<0.001***	1.658–2.128	1.879	<0.001***

DIS, difficulty initiating sleep; DMS, difficulty maintaining sleep; EMA, early morning awakening. PSS-4, Perceived Stress Scale 4. *p < 0.05, ***p < 0.001.

a CI, Confidence Interval.

b China’s professional title system for nurses is hierarchical, consisting of three levels and five titles, with progressively increasing requirements for academic degree and working experience at each level. Primary title includes Nurse and Nurse Practitioner; Intermediate title includes Supervisor Nurse (Nurse in charge); High title includes Associate Chief Nurse and Chief Nurse.

#### 3.3.1 Demographic variables

**Figure 2:**
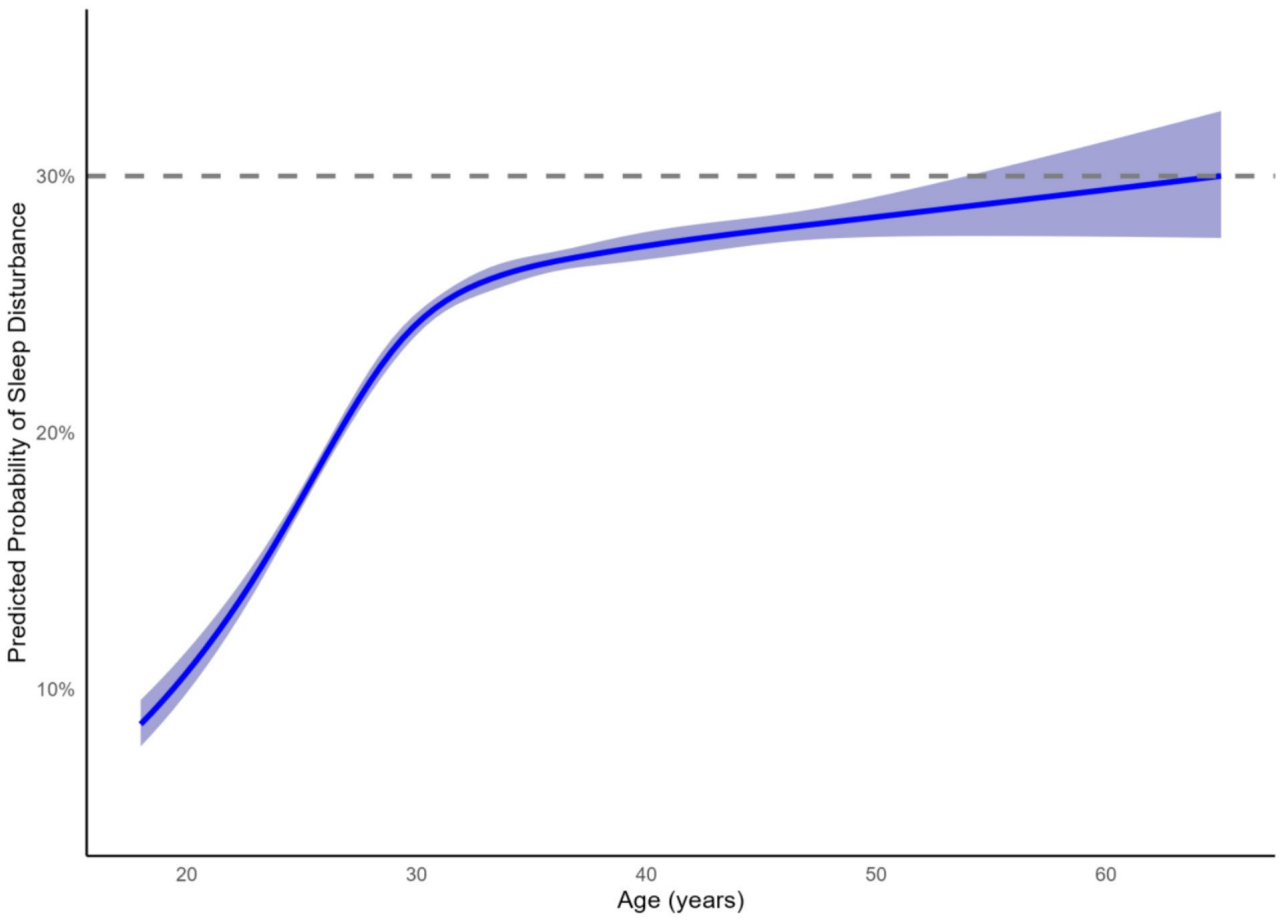
Association between age and predicted probability of sleep disturbance among nurses using restricted cubic spline regression. The solid blue line represents the predicted probability of sleep disturbance derived from a restricted cubic spline model with 5 knots; the shaded area indicates the 95% confidence interval.

With respect to age, compared to the reference group (<30 years), nurses aged 30–39 years had an increased risk of sleep disturbance (aOR = 1.226, 95%CI: 1.167–1.288) and its sub-types (DIS: aOR = 1.195, 95%CI: 1.137–1.256; DMS: aOR = 1.448, 95%CI: 1.362–1.540; EMA: aOR = 1.248, 95%CI: 1.176–1.325). The risk was even higher for nurses≥40 years (sleep disturbance: aOR = 1.427, 95%CI: 1.339–1.520; DIS: aOR = 1.448, 95%CI: 1.362–1.540; DMS: aOR = 1.844, 95%CI: 1.713–1.984; EMA: aOR = 1.543, 95%CI: 1.435–1.659). Further exploration using restricted cubic splines delineated a non-linear age-dependent risk pattern (Figure 2). The predicted probability rose sharply in early adulthood, plateaued during midlife, and ascended again gradually in later adulthood. Consistent with multivariable regression results, Generalized Additive Models provided detailed insight into these associations.

Females exhibited a higher risk of sleep disturbance compared to males (aOR = 1.400, 95%CI: 1.316–1.489), with consistent elevated risks across sub-types (DIS: aOR = 1.353, 95%CI: 1.271–1.441; DMS: aOR = 1.708, 95%CI: 1.002–1.160; EMA: aOR = 1.510, 95%CI: 1.397–1.631). Separated, widowed or divorced marital status increased sleep disturbance risk (aOR = 1.276, 95%CI: 1.174–1.386) and its sub-types (DIS: aOR = 1.208, 95%CI: 1.108–1.316; DMS: aOR = 1.295, 95%CI: 1.175–1.427; EMA: aOR = 1.368, 95%CI: 1.241–1.507).

#### 3.3.2 Occupational variables

Regarding working experience, compared to the reference group (<5 years), nurses with 5–9 years of experience had elevated risks of sleep disturbance (aOR = 1.334, 95%CI: 1.272–1.399) and sub-types (DIS: aOR = 1.323, 95%CI: 1.260–1.389; DMS: aOR = 1.275, 95%CI: 1.199–1.355; EMA: aOR = 1.296, 95%CI: 1.221–1.376). Nurses with ≥10 years of experience showed further increased risks (sleep disturbance: aOR = 1.507, 95%CI: 1.416–1.592; DIS: aOR = 1.502, 95%CI: 1.416–1.592; DMS: aOR = 1.565, 95%CI: 1.457–1.681; EMA: aOR = 1.478, 95%CI: 1.378–1.585). night shift work was a risk factor (aOR = 1.231, 95%CI: 1.192–1.271), with consistent effects across sub-types.

Protective factors included a high job title [Associate Chief Nurse/Chief Nurse; reference: Primary title (Nurse/Nurse Practitioner)] (aOR = 0.863, 95%CI: 0.802–0.928) and higher education (bachelor’s degree or above: aOR = 0.571, 95%CI: 0.524–0.623 vs. secondary/vocational education).

#### 3.3.3 Lifestyle variables

Napping showed protective effects: napping 30–60 min (aOR = 0.661, 95%CI: 0.641–0.681) and >60 min (aOR = 0.662, 95%CI: 0.627–0.699) reduced sleep disturbance risk, compared to no napping. Risk factors included smoking (current smoking: aOR = 1.325, 95% CI: 1.226–1.431; used to smoke: aOR = 1.860, 95% CI: 1.543–2.243) and alcohol use (drinking ≥2/week: aOR = 2.204, 95% CI: 1.977–2.456).

## 4 Discussion

### 4.1 Principal findings

To our knowledge, this is the first multi-center survey after COVID-19 concerning sleep disturbances and associated risk factors of nurses in Mainland China. This study provides an updated and reliable prevalence of sleep disturbance at 24.1% based on baseline data from the NHMS. The results show that female nurses, night shift workers, smokers, and drinkers are more likely to develop sleep disturbance, whereas those who hold senior positions and participate in regular physical activity are less likely to suffer from sleep disturbance. The findings provide a reference for the development of targeted interventions to improve the well-being and performance of nurses.

### 4.2 Conservative prevalence among nurses in public hospitals in China

A notable finding is that the overall prevalence of sleep disturbances was 24.1%, significantly lower than previous studies (11, 12). This number was 29.6% in the US (33), 42.2% in Sweden (34), 76% in France (35), 49.9% in China (13), 69.8% in Malaysia (36), 41.2% in Japan (37) and 75.5% in Ethiopia (10).

The variation can be attributed to three key factors: firstly, this study defined sleep disturbances using a more conservative criterion (“often” experiencing specific symptoms), differing from binary assessments or scales like the PSQI used in previous research (36). This methodological choice is supported by a large-scale study employing comparable symptom-frequency criteria, which documented an overall prevalence of 22.1% (31). Furthermore, the prevalence rates for the individual subtypes in our data demonstrate notable consistency with that report: difficulty initiating sleep (13.3% vs. 14.3%); difficulty maintaining sleep (15.5% vs. 16.2%); and early morning awakening (15.4% vs. 12.4%). This strong concordance suggests that our stringent definition effectively identified a population with clinically relevant sleep disturbance symptoms. Secondly, the study focused exclusively on nurses in tertiary public hospitals. These institutions typically feature more structured work environments, scientific shift scheduling, and comprehensive employee wellness programs, which may collectively mitigate the severity and prevalence of sleep issues compared to broader settings. Thirdly, the potential for social desirability bias, which is common in self-reported health surveys within professional settings, may have led to a conservative estimation (underreporting) of the true prevalence of sleep disturbances.

### 4.3 High-risk groups for sleep disturbances among nurses

Our findings reveal several high-risk groups for sleep disturbances among nurses: female nurses, night shift workers, individuals aged 30 to 39 years, those with low education levels and intermediate professional titles, non-nappers, smokers and drinkers are particularly vulnerable. Consensus with prior research highlights that female nurses and those over 30 exhibit higher sleep disturbance rates (38). Notably, our analysis revealed a significant non-linear relationship between age and sleep risk, suggesting that the underlying drivers of sleep disturbance evolve across the career lifespan: from adaptation stress in early career, to persistent work–family conflict throughout midlife, and finally to age-related physiological changes in later years (14, 39, 40).

The refined age-dependent pattern provides specific guidance for interventions. The finding that high night shift exposure strongly intensifies the age-related increase in sleep disturbance risk, particularly among nurses aged 30–50, indicates that mid-career nurses with high night shift exposure are a critical subgroup for targeted intervention and occupational health support. Moreover, the distinct age-related patterns observed, particularly the sharp rise and high prevalence of Difficulty Maintaining Sleep (DMS) across much of the working age, underscores the importance of DMS as a central age-related sleep issue within this nursing population.

We posit that female nurses may be more susceptible due to hormonal fluctuations and societal roles (39). In China, cultural norms place significant demands on women aged 30–39 to balance marriage, childbirth and child-rearing while pursuing professional advancement. This dual burden creates work–family conflict, increasing sleep disturbances (41). Therefore, our findings advocate for targeted intervention strategies: structural support (such as flexible scheduling and childcare assistance) may prove more effective for middle-aged nurses in the plateau phase, while older nurses may benefit more from individual-level guidance like sleep hygiene education.

Some studies report poorer sleep quality among male ICU nurses due to physical demands and higher sleep disturbance prevalence among younger nurses (<30 years) due to rotation, interpersonal, and career pressures; however, these findings are largely mediated by job stress (14, 40). Notably, job stress surged during COVID-19, sharply increasing acute insomnia among nurses, and despite the pandemic’s end, persistent stress continues to drive global nursing workforce attrition (3, 42)^.^ In China, for example, the large population and low nurse-to-patient ratios (1,8.0 during day shifts; 1:23 at night) further exacerbate workloads (43). To effectively manage nurse stress, two evidence-based interventions are recommended for hospitals: (1) Implement dynamic workload allocation that considers individual factors, including physiological capacity, family obligations, and career development needs. For instance, younger nurses with less experience could be paired with senior staff during high-stress shifts. (2) Establish standardized staffing models based on patient acuity levels, ensuring that critical care units maintain optimal NTP ratios (e.g., 1:4 in ICUs) to reduce burnout risks.

### 4.4 Impact of night shift work on sleep disturbances

In accordance with global evidence, night shift work has been demonstrated to increase the risk of sleep disturbance via circadian disruption (44). Shift systems have different effects on nurses’ work performance and occupational health (45). For example, the U.S. practice of long-term fixed night shifts minimizes circadian disruption but correlates with low job satisfaction, whereas rapid-rotating shifts in China, South Korea and Iran—while enhancing workforce flexibility—exacerbate sleep fragmentation and medical error risks (46, 47). These cross-cultural differences call for evidence-based shift design studies that balance physiological needs with work requirements. A study among 50 Italian nurses revealed that nurses working backward shifts experienced a higher prevalence of sleep disturbances and work-life imbalances compared to their forward-shift counterparts (48). The Dutch PerfectFit@Night program, which combines circadian-adaptive lighting, sleep hygiene education and personalized shift scheduling, has been proved to reduce sleep disturbances (49). This model could be adapted in collectivist settings like China by integrating group-based support to address cultural preferences for communal solutions, contrasting with individual-focused approaches in the U.S. Additionally, designating a “post-night shift sleep day” offers a scalable strategy to mitigate cumulative sleep debt, particularly relevant for countries with high nurse-to-patient ratios.

### 4.5 Association of education level and department with sleep disturbances

The PerfectFit@Night intervention incorporates e-learning modules and sleep coaching at the individual level, aiming to enhance nurses’ sleep quality and coping skills (49)^.^ This objective aligns with our findings that nurses with higher education levels tend to experience lower rates of sleep disturbances, as educated nurses demonstrate greater psychological resilience and resource utilization for stress management (50). Therefore, education about sleep health should also be included in employee care programs.

For departments, this study identified emergency medicine, ICUs, pediatrics and obstetrics as high-prevalence departments for nurse sleep disturbances, consistent with global research showing these units experience intense patient acuity and workflow complexity (51, 52). The heightened prevalence in large departments like obstetrics may be attributed to the substantial patient caseload, complexity of medical conditions and environmental stressors (53). However, in contrast to previous studies, the rate of sleep disturbance among psychiatric nurses in this study was low (40). It may be a result of the low percentage of the department and the relatively small sample size. This is also supported by the large standard error (SE = 1.3). Special units such as ICUs and Emergency departments also exhibited elevated prevalence, likely influenced by the intense, urgent, and critical nature of nursing duties (54, 55)^.^ Departments with elevated prevalence rates should receive enhanced attention, such as additional nursing staff and double shifts at night (56). Targeted interventions can be implemented based on departmental characteristics. For example, obstetrics departments can optimize flexible scheduling and assign double the number of midwives during peak delivery periods; emergency departments can deploy AI triage systems to assist with manual triage; and ICUs can turn off non-essential alarms during daily lunch breaks.

### 4.6 Lifestyle factors affecting sleep disturbances among nurses

Due to the influence of the human biological clock and circadian rhythm, the ability to remain fully awake is limited (57). Napping aids nurses in recovering from burnout and sleepiness (45). However, studies indicate that high workloads prevent many nurses from taking short breaks (naps) and obtaining sufficient rest between shifts (58). When nurses lack adequate rest, the risk to patient safety significantly increases (59). It is recommended that the scheduling system include nap time in the work routine of shift nurses. Alcohol and cigarettes have contrasting effects on mental activity but both negatively impact long-term sleep quality (60, 61).

Current intervention measures primarily target organizational factors. However, emerging evidence emphasizes the necessity of developing profession-specific exercise prescriptions for nurses’ sleep health management. Integration of moderate aerobic exercise, light strength exercise and mind–body exercise was more effective in improving sleep quality of shift nurses (62). Future research should prioritize: (1) tailoring exercise modalities to clinical specialty characteristics (e.g., low-impact yoga for obstetric nurses who stand for prolonged periods; high-intensity interval training for intensive care unit nurses working 12-h shifts); (2) culturally adapting interventions to national exercise guidelines; (3) Strict validation should utilize wearable sleep-exercise synchronized monitoring devices to establish a dose–response relationship between exercise patterns and improvements in sleep structure.

In conclusion, this study offers valuable insights into the correlates of sleep disturbances among nurses and serves as a significant reference for future research and health policy development. However, the factors influencing sleep disturbances in nurses differ based on individual fitness, socio-cultural background, and healthcare system (63). Therefore, international scholars should consider the findings of this study in conjunction with the specific contexts of their respective countries.

### 4.7 Limitations

This study has several limitations. Firstly, the data relied on self-reported information, which may have introduced recall and self-reported biases, including the potential for social desirability bias in self-reported lifestyle factors (smoking, drinking) and sleep outcomes. To mitigate these biases, future research should employ objective measures. Secondly, the cross-sectional design used in this study limits the ability to establish causality between sleep disturbance and its correlates. Future studies should adopt longitudinal designs to explore these causal relationships. Thirdly, the generalizability of the findings is restricted as the sample was drawn exclusively from tertiary public hospitals. Consequently, the results may not apply to all nurses in China, particularly those working in rural settings. Fourthly, the study’s use of self-reported criteria to define sleep disturbance as a singular dimension may not fully capture its multifaceted nature. Future research could enhance validity by incorporating additional dimensions such as PSQI. Fifthly, the study did not capture detailed shift characteristics, such as rapid versus slow rotation, which introduces the potential for residual confounding related to complex shift work patterns.

## 5 Conclusion

The significant prevalence of sleep disturbances among nurses in China demands urgent attention. Female nurses aged 30 to 39, holding intermediate titles with secondary or higher vocational education and engaging in unhealthy lifestyle habits such as smoking, drinking, and lack of exercise, face a heightened risk of sleep disturbance. It is imperative for nurses to proactively manage and regulate their well-being, while managers must earnestly consider strategies for identifying and intervening with high-risk groups.

## Data Availability

The raw data supporting the conclusions of this article will be made available by the authors, without undue reservation.

## Footnotes

1
www.wjx.cn
