# Supplementary material for: Prevalence of sleep disturbance and associated factors among nurses in Chinese tertiary public hospitals: a national cross-sectional study
Source: Front Public Health. 2026 Jan 21;13:1735543. doi: 10.3389/fpubh.2025.1735543 (PMC12871391; doi:10.3389/fpubh.2025.1735543)
Supplement: Supplementary file 1 [file Table_1.docx]

Prevalence of Sleep Disturbance and Associated Factors among Chinese Nurses: A Cross-sectional Study

| **Table S1** Prevalence of Sleep Disturbance by department (N=132910) | | | | | | | | | | | | | | | |
| --- | --- | --- | --- | --- | --- | --- | --- | --- | --- | --- | --- | --- | --- | --- | --- |
| Department | No Sleep Disturbance  (n=100933) | | | Sleep Disturbance  (n=31977) | | | DIS  (n=17721) | | | DMS  (n=20605) | | | EMA  (n=20445) | | |
|  | n | % | SE | n | % | SE | n | % | SE | n | % | SE | n | % | SE |
| Internal medicine | 26354 | 76.3 | 0.2 | 8165 | 23.7 | 0.2 | 4617 | 13.4 | 0.2 | 5273 | 15.3 | 0.2 | 5172 | 15.0 | 0.2 |
| Surgery | 22304 | 76.1 | 0.2 | 7024 | 23.9 | 0.2 | 3869 | 13.2 | 0.2 | 4565 | 15.6 | 0.2 | 4402 | 15.0 | 0.2 |
| Gynecologic | 4971 | 73.6 | 0.5 | 1781 | **26.4** | 0.5 | 959 | **14.2** | 0.4 | 1150 | **17.0** | 0.5 | 1143 | **16.9** | 0.5 |
| Ear, Nose and Throat (ENT) | 3208 | 77.1 | 0.7 | 954 | 22.9 | 0.7 | 492 | 11.8 | 0.5 | 607 | 14.6 | 0.5 | 633 | 15.2 | 0.6 |
| Pediatrics | 3328 | 74.3 | 0.7 | 1150 | **25.7** | 0.7 | 625 | **14.0** | 0.5 | 763 | **17.0** | 0.6 | 730 | **16.3** | 0.6 |
| Psychiatry | 763 | 82.2 | 1.3 | 165 | 17.8 | 1.3 | 102 | 11.0 | 1.0 | 99 | 10.7 | 1.0 | 79 | 8.5 | 0.9 |
| Department of Infectious Diseases | 1548 | 75.6 | 0.9 | 499 | 24.4 | 0.9 | 284 | 13.9 | 0.8 | 303 | 14.8 | 0.8 | 318 | 15.5 | 0.8 |
| Intensive Care Unit (ICU) | 11379 | 74.1 | 0.4 | 3985 | **25.9** | 0.4 | 2304 | **15.0** | 0.3 | 2498 | **16.3** | 0.3 | 2524 | **16.4** | 0.3 |
| Outpatient | 4928 | 75.7 | 0.5 | 1578 | 24.3 | 0.5 | 845 | 13.0 | 0.4 | 1082 | **16.6** | 0.5 | 1145 | **17.6** | 0.5 |
| Emergency | 5913 | 73.5 | 0.5 | 2136 | **26.5** | 0.5 | 1252 | **15.6** | 0.4 | 1370 | **17.0** | 0.4 | 1357 | **16.9** | 0.4 |
| Operating room | 7784 | 78.9 | 0.4 | 2076 | 21.1 | 0.4 | 1022 | 10.4 | 0.3 | 1303 | 13.2 | 0.3 | 1304 | 13.2 | 0.3 |
| Department of Nursing | 1195 | 78.9 | 1.0 | 319 | 21.1 | 1.0 | 183 | 12.1 | 0.8 | 200 | 13.2 | 0.9 | 210 | 13.9 | 0.9 |
| Other | 7258 | 77.2 | 0.4 | 2145 | 22.8 | 0.4 | 1167 | 12.4 | 0.3 | 1392 | 14.8 | 0.4 | 1428 | 15.2 | 0.4 |
| Total | 100933 | 75.9 | 0.1 | 31977 | **24.1** | 0.1 | 17721 | **13.3** | 0.1 | 20605 | **15.5** | 0.1 | 20445 | **15.4** | 0.1 |

DIS, difficulty initiating sleep; DMS, difficulty maintaining sleep; EMA, early morning awakening. SE: Standard Error.

**Table S2.** Summary of Questionnaire Survey Data on Nurses from 67 Tertiary Public Hospitals in China

| Hospital | Valid* Respondents(n) | Respondents(n) | Total nurse(n) | Response Rate (%) | |
| --- | --- | --- | --- | --- | --- |
| Peking Union Medical College Hospital | 1972 | 1985 | 2242 | 88.54 | |
| Peking University First Hospital | 2006 | 2013 | 2258 | 89.15 | |
| Xiangya Second Hospital of Central South University | 2704 | 2752 | 2840 | 96.90 | |
| Xiangya Hospital of Central South University | 2314 | 2355 | 2700 | 87.22 | |
| Hunan Provincial People's Hospital | 2079 | 2094 | 2690 | 77.84 | |
| Tianjin Medical University General Hospital | 1439 | 1448 | 1600 | 90.50 | |
| Tianjin Third Central Hospital | 1079 | 1087 | 1140 | 95.35 | |
| Shanghai Tenth People's Hospital | 1249 | 1249 | 1258 | 99.28 | |
| Ninth People's Hospital, Affiliated to Shanghai Jiao Tong University School of Medicine | 1866 | 1871 | 2105 | 88.88 | |
| Second Affiliated Hospital of Army Medical University | 1716 | 1718 | 1851 | 92.81 | |
| First Affiliated Hospital of Chongqing Medical University | 1936 | 1949 | 2045 | 95.31 | |
| First Affiliated Hospital of Harbin Medical University | 2653 | 2770 | 2781 | 99.60 | |
| Second Affiliated Hospital of Harbin Medical University | 1992 | 2013 | 2176 | 92.51 | |
| Third Hospital of Jilin University | 2275 | 2507 | 2462 | 101.83 | |
| First Hospital of Jilin University | 3209 | 3286 | 3544 | 92.72 | |
| First Affiliated Hospital of Dalian Medical University | 1863 | 1886 | 2060 | 91.55 | |
| Second Affiliated Hospital of Dalian Medical University | 1814 | 1836 | 2097 | 87.55 | |
| First Affiliated Hospital of Hebei Medical University | 1096 | 1100 | 1202 | 91.51 | |
| Hebei Provincial People's Hospital | 892 | 898 | 936 | 95.94 | |
| First Affiliated Hospital of Zhengzhou University | 5155 | 5275 | 6087 | 86.66 | |
| Henan Provincial People's Hospital | 3253 | 3265 | 3599 | 90.72 | |
| Gulou Hospital, Affiliated to Nanjing University School of Medicine | 2565 | 2582 | 2936 | 87.94 | |
| Zhongda Hospital, Affiliated to Southeast University | 1448 | 1482 | 1688 | 87.80 | |
| Shandong Provincial Hospital | 3055 | 3105 | 3138 | 98.95 | |
| Affiliated Hospital of Qingdao University | 3094 | 3103 | 3540 | 87.66 | |
| First Affiliated Hospital of Anhui Medical University | 2115 | 2115 | 2018 | 104.81 | |
| Hefei First People's Hospital | 1619 | 1648 | 1724 | 95.59 | |
| First Affiliated Hospital of Shanxi Medical University | 1442 | 1472 | 1666 | 88.36 | |
| Bethune Hospital, Shanxi | 1760 | 1794 | 1836 | 97.71 | |
| Shaanxi Provincial People's Hospital | 1782 | 1835 | 2096 | 87.55 | |
| First Affiliated Hospital of Xi'an Jiaotong University | 2589 | 2636 | 2956 | 89.17 | |
| Second Hospital of Lanzhou University | 2033 | 2098 | 2373 | 88.41 | |
| Jiuquan People's Hospital, Gansu | 722 | 734 | 833 | 88.12 | |
| West China Hospital, Sichuan University | 3663 | 3724 | 4158 | 89.56 | |
| Sichuan Provincial People's Hospital | 1745 | 1766 | 1981 | 89.15 | |
| Affiliated Hospital of Southwest Medical University | 1883 | 1906 | 2031 | 93.85 | |
| Qinghai Provincial People's Hospital | 1488 | 1532 | 1611 | 95.10 | |
| Qinghai Red Cross Hospital | 1230 | 1234 | 1300 | 94.92 | |
| Tongji Hospital, Affiliated to Huazhong University of Science and Technology | 4090 | 4192 | 4355 | 96.26 | |
| Wuhan Central Hospital | 2399 | 2466 | 2493 | 98.92 | |
| Jiangxi Provincial People's Hospital | 1345 | 1380 | 1472 | 93.75 | |
| First Affiliated Hospital of Nanchang University | 3303 | 3330 | 3801 | 87.61 | |
| Second Affiliated Hospital of Nanchang University | 1884 | 1897 | 2186 | 86.78 | |
| Sir Run Run Shaw Hospital, Zhejiang University | 2467 | 2477 | 2694 | 91.95 | |
| Second Affiliated Hospital of Zhejiang University School of Medicine | 2253 | 2304 | 2387 | 96.52 | |
| First Affiliated Hospital of Zhejiang University School of Medicine | 2525 | 2535 | 2619 | 96.79 | |
| Union Hospital, Fujian Medical University | 1854 | 1867 | 1955 | 95.50 | |
| Fujian Provincial General Hospital | 2196 | 2211 | 2258 | 97.92 | |
| Guizhou Provincial People's Hospital | 966 | 971 | 1064 | 91.26 | |
| Affiliated Hospital of Zunyi Medical University | 1908 | 1909 | 1805 | 105.76 | |
| Guangdong Provincial People's Hospital | 1609 | 1629 | 2266 | 71.89 | |
| Nanfang Hospital, Southern Medical University | 2228 | 2268 | 2796 | 81.12 | |
| Fifth Affiliated Hospital of Sun Yat-sen University | 1210 | 1223 | 1467 | 83.37 | |
| First Affiliated Hospital of Kunming Medical University | 2229 | 2280 | 2497 | 91.31 | |
| Yunnan Provincial First People's Hospital | 1772 | 1794 | 2038 | 88.03 | |
| Hainan Provincial People's Hospital | 2155 | 2155 | 2218 | 97.16 | |
| First Affiliated Hospital of Hainan Medical College | 1110 | 1120 | 1180 | 94.92 | |
| First Affiliated Hospital of Guangxi Medical University | 1511 | 1518 | 1726 | 87.95 | |
| Guangxi Zhuang Autonomous Region People's Hospital | 1858 | 1886 | 2185 | 86.32 | |
| First Affiliated Hospital of Xinjiang Medical University | 2071 | 2087 | 2373 | 87.95 | |
| Xinjiang Uygur Autonomous Region People's Hospital | 1992 | 2024 | 2227 | 90.88 | |
| Affiliated Hospital of Inner Mongolia Medical University | 1861 | 1870 | 2018 | 92.67 | |
| Inner Mongolia Autonomous Region People's Hospital | 1718 | 1746 | 1983 | 88.05 | |
| Ningxia Medical University General Hospital | 2228 | 2317 | 2624 | 88.30 | |
| Ningxia Hui Autonomous Region People's Hospital | 648 | 650 | 739 | 87.96 | |
| Tibet Autonomous Region People's Hospital | 464 | 472 | 530 | 89.06 | |
| Shigatse People's Hospital, Tibet | 261 | 272 | 318 | 85.53 | |
| Total | 132910 | 134973 | 147832 | 91.30 | |
| *Valid refers to responses that are considered to meet the criteria for analysis after data cleaning. | | | | |  |
