# Supplementary material for: Prevalence of sleep disturbance and associated factors among nurses in Chinese tertiary public hospitals: a national cross-sectional study
Source: Front Public Health. 2026 Jan 21;13:1735543. doi: 10.3389/fpubh.2025.1735543 (PMC12871391; doi:10.3389/fpubh.2025.1735543)
Supplement: Supplementary file 2 [file Data_Sheet_1.docx]

**Nurses’ Mental Health Study (NMHS)**

**Ethical approval number：LYF20230048**

**Trial registration number：ChiCTR2300072142**

Dear Nursing Colleagues,

Greetings! The Chinese Nursing Association extends heartfelt appreciation for your hard work!

As crucial contributors on the frontlines of medical and healthcare, you, the guardians of people's health, bear significant physical and psychological pressures while safeguarding lives and well-being.

The Chinese Nursing Association is steadfast in its care for each nurse, dedicated to researching and exploring strategies to enhance nurses' well-being and happiness. In this regard, we warmly invite you to participate in a health survey designed specifically for nurses. Your authentic feedback is invaluable; it will serve as evidence supporting the establishment of a comprehensive nurse health service system. Moreover, it will provide a scientific foundation for the government to formulate effective prevention and control policies addressing nurses' health issues, ultimately working towards practical and reliable benefits for all.

Believe that your participation and dedication will echo as a robust voice advocating for the health of both yourself and your fellow nursing colleagues! The Chinese Nursing Association stands in solidarity with you every step of the way.

Best Regards,

Wu Xinjuan

Chinese Nursing Association

**Nurses’ Mental Health Study (NMHS)–Questionnaire**

| **This questionnaire comprises general information and inquiries pertaining to occupation and health. Kindly complete it attentively based on your experience. There are no right or wrong answers. If uncertain about a response, please select the option closest to your genuine thoughts. Thank you!** | |
| --- | --- |
| **Section 1. Sociodemographic Information** | |
| **1.1 Gender：** | □Male □Female |
| **1.2 Date of birth：** | (Year) (Month) |
| **1.3 Ethnicity：** | □Han □Minority（Fill in the full name of your ethnicity） |
| **1.4 Height：** | cm |
| **1.5 Weight：** | Kg |
| **1.6 Years of working：** | Years（Please fill in integers） |
| **1.7 Title：** | □Junior □Mid-level □Senior |
| **1.8 Position：** | □None □ Head Nurse/Head Nurse of Department □ Director/Deputy Director of Nursing |
| **1.9 Education level：** | □ Associate degree or below □Bachelor □Master □Doctor |
| **1.10**  **Your current marital status ：**  □ Single **→**If you choose this option, Answer **1.10b**  □ Married **→**If you choose this option, Answer **1.10a、1.10b、1.10c、1.10d、1.10e**  □ Divorced **→**If you choose this option, Answer **1.10b、1.10c**  □ Remarried **→** If you choose this option, Answer **1.10a、1.10b、1.10c、1.10d、1.10e**  □ Widowed **→**If you choose this option, Answer **1.10b、1.10c**  **1.10a Is your spouse a healthcare work？**  □ No □ Yes **→**If you choose this option, Please tick the follows：□Doctor □Nurse □Others (Medical technician)  **1.10b Are you pregnant？（If you choose “Female” at question 1.1）**  □No □Yes **→**If you choose this option, Please fill in the blank **pregnant_______ months skip to 1.11**  **Your maternity history：** **pregnant________** **giving birth_________ （Please fill in integers）**  **1.10c Do you have children？**  □ No **→**If you choose this option, Answer **1.10d**  □ 1 **→**If you choose this option, Answer **1.10e**  □ 2 **→**If you choose this option, Answer **1.10e**  □ 3 or more **→**If you choose this option, skip to **1.11**  **1.10d Do you have pregnancy planning？**  □ No **→**If you choose this option, Answer **1.10f**  □ Yes, plan to have a child in 1 year  □ Yes, yet not in 1 year **→**If you choose this option, Answer **1.10g**  **1.10e Do you have a plan to have more children？**  □No **→**If you choose this option, Answer **1.10f**  □Yes, plan to have a child in 1 year  □Yes, yet not in 1 year **→**If you choose this option, Answer **1.10g**  **1.10f. Reason for not having a child at present**  □ Enough children at present  □ Financial constraints  □ Lack of person to care children  □ Harm career development  □ Physical causes  □ Other  **1.10g. Reason for not having a child at present**  □ Had fertility experience recently  □ Financial constraints  □ Lack of person to care children  □ Harm career development  □ Physical causes  □ Other | |
| **1.11 Do you have religious belief？**  □No □Yes **→**If you choose this option, Answer **1.11a、1.11b、1.11c**  **1.11a What is your religion？？**  □Buddhism □Christianism □Islam □Other  **1.11b How long have you been practicing the religion Years？（Please fill in integers）**  **1.11c *In the past year*, how often do you attend religious activities？**  □ Rarely  □ ＜1 time/month  □ 1~3 times/month  □ 1 time/week  □ ＞1 time/week | |

| **Section 2.** **Work-related Information** |
| --- |
| **2.1  *In the past year*, your main responsibility is：**□Direct patient care □Non-direct patient care |
| **2.2 Your current unit/department：**  □Internal Medicine □Surgery Department □Obstetrics and Gynecology Department □ENT & Ophthalmology Department □Pediatrics Department □Psychiatry Department □Infectious Diseases Department □Intensive Care Unit (ICU) □Outpatient Department □Emergency Department □Operating Room (OR) □Nursing Department □Others |
| **2.3 *In the past year,* how many hours do you work in hospital in a week on average？** hrs.**（Please fill in integers）** |
| **2.4 *In the past year,* do you work in night shifts？（Work between 0:00 to 6:00 for more than 3h）**  □No  □Yes **→**If you choose this option, Answer **2.4a**  **2.4a *In the past year,* how many night shifts did you take in a month on average? （Please fill in integers）** |
| **2.5 Till now, how long have you worked in night shifts**  years**（Please fill in integers）（**Night shift work is defined as working for at least 3 hours from 0: 00 to 6: 00 and for at least 3 night shifts per month.**）** |
| **Answer 2.6、2.7, if you choose “clinical work”** |
| **2.6 *In the past year,* you are responsible for patient(s) in day shifts; you are responsible for patient(s) in middle shifts; you are responsible for patient(s) in night shifts normally（Answer this question, if you choose “clinical work” in 2.1 and choose “Yes” in 2.4）**  **2.6 *In the past year,* you are responsible for patient(s) in day shifts; you are responsible for patient(s) in middle shifts normally（Answer this question, if you choose “clinical work” in 2.1 and choose “No” in 2.4）**  **Note: If you don't directly care for patients, please fill in "0".** |
| **2.7 How much paperwork is going on your daily routine？**  □ Very easy □ Relatively easy □ Fair □ Relatively heavy □ Very heavy |
| **2.8 How well do you balance work and life？**□Very good □Good □Fair □Poor □Very poor |
| **2.9 *In the past year,* have you been involved in the following events? (if you choose “clinical work” in 2.1）**  2.9a Patient falls □No □Yes  2.9b Patient missing □No □Yes  2.9c Patient suicides □No □Yes  2.9d Patient deaths □No □Yes  2.9e Patients or Families complaints □No □Yes  2.9f Nursing-related adverse events (caused by errors in the execution of medical orders, improper nursing operations, etc., yet without serious consequences)  □No □Yes  2.9g Medical malpractice (serious adverse consequences due to errors in executing medical orders or improper nursing operations, such as disability, damage to tissues and organs, etc.)  □No □Yes  2.9h Needlestick and sharps injuries (NSIs)  □No □Yes **→**If you choose this option, Answer this: Did you prickled by needles contaminated with HIV, Syphilis or Hepatitis B  2.9i Receive a thank-you letter/pennant from the patient or family member you supervised □No □Yes |
| **2.10 Workplace violence**  **2.10a *In the past year,* do you have the following experience in the workplace?**  1.Physical assault  □None □1 time □2-3 times □>3 times □ Prefer not to answer  2.Emotional abuse (disrespect and disparagement words)  □None □1 time □2-3 times □>3 times □ Prefer not to answer  3.Threats  □None □1 time □2-3 times □>3 times □ Prefer not to answer  4.Verbal sexual harassment (unwelcome remarks or comments of a sexual nature)  □None □1 time □2-3 times □>3 times □ Prefer not to answer  5.Sexual abuse (unwanted touching or other sexual behaviors)  □None □1 time □2-3 times □>3 times □ Prefer not to answer  **2.10b**  **In the past year, have you ever encountered workplace** **bullying？** (repeatedly isolated, work hard belittled, threatened, talked about, etc.)  □Never  □Several times  □Sometimes  □Often  □Always  □Prefer not to answer |
| **2.11 Are you satisfied with the current atmosphere of your workplace？**  □Very satisfied □Relatively satisfied □Fair □Relatively not satisfied □Extremely not satisfied |
| **2.12** **Do you think your work is meaningful?**  □Very meaningful □Meaningful □Fair □Meaningless □Very meaningless |
| **2.13 How do you feel about your career development?** □Very good □Good □Fair □Poor □Very poor |
| **2.14 If you could choose again, would you still be a nurse?** □Yes □No |
| **2.15 Would you like your children to work as nurses?** □Yes □No |
| **2.16 Intent to leave**  **2.16a *In the past year,* how many times have you thought about leaving?**  □Never  □Seldom  □Sometimes  □Often **→**If you choose this option, Answer **2.16b、2.16c**  □Prefer not to answer  **2.16b When you are considering leaving, what do you plan to do?**  □Take a break □Get further education □Working as a nurse in other institutions  □ Go to work in other industries □ No longer employed □Not clear yet  **2.16c Main reasons for your turnover [Multiple selections, no more than three items]**  □Night shifts □ Burnout □ Physical causes □ Heavy work stress  □Difficult to take care of the family □Family not support □ Low welfare package  □High occupational risk □Poor interpersonal relationships □ Others (please specify) |
| **Answer 2.17、2.18：if 1.8 choose“no”and 2.1 choose“clinical work”** |
| **2.17 The extent to which you agree with the following statements, your head nurse is fair and just in shift scheduling, etc.**  □ Totally agree □Agree □Fair □ Disagree □ Totally disagree |
| **2.18 The extent to which you agree with the following statements, your head nurse cares about you.**  □ Totally agree □Agree □Fair □ Disagree □ Totally disagree |

| **Section 3. Health status** | | | | | | | | | | | |
| --- | --- | --- | --- | --- | --- | --- | --- | --- | --- | --- | --- |
| **3.1 *In the past year,* do you often feel fatigue?**  □Always □Often □Sometimes □Seldom □Never | | | | | | | | | | | |
| **3.2 *In the past year,* do you often feel lonely?** □No □Yes | | | | | | | | | | | |
| **3.3 When things change, are you able to adapt?**  □Always □Often □Sometimes □Seldom □Never | | | | | | | | | | | |
| **3.4 After an illness or distress, is it easy for you to adjust yourself?**  □Always □Often □Sometimes □Seldom □Never | | | | | | | | | | | |
| **3.5 Using your own definition of “burnout”, please choose one of the answers below:**  □I enjoy my work. I have no symptoms of burnout  □I am under stress, and don't always have as much energy as l did, but I don't feel burned out  □I am beginning to burn out and have one or more symptoms of burnout, e.g. emotional exhaustion  □The symptoms of burnout that I am experiencing won’t go away. I think about work frustrations a lot  □I feel completely burned out. l am at a point where I may need to seek help | | | | | | | | | | | |
| **3.6 *Over the past 12 months,* have you thought about committing suicide?**  □No □Yes □ Prefer not to answer  **3.7 *In the past year,* did you practice to harm yourself?**  □No □Yes **→**If you choose this option, Answer **3.7a** □ Prefer not to answer  **3.7a Did you harm yourself to take your own life?**  □No □Yes □ Prefer not to answer | | | | | | | | | | | |
| **3.8 *In the past 1 month*, how often do you experience the following events?**   ***PSS-4***  1. You were unable to control the important things in your life  □Never □Almost never □Sometimes □Fairly often □Very often  2. You felt confident about your ability to handle your personal problems  □Never □Almost never □Sometimes □Fairly often □Very often  3. You felt that things were going your way  □Never □Almost never □Sometimes □Fairly often □Very often  4. You felt difficulties were piling up so high that you could not overcome them  □Never □Almost never □Sometimes □Fairly often □Very often | | | | | | | | | | | |
| **3.9 In the past 2 weeks, how often do you experience the following events? PHQ-9 Depression** | | | | | | | | | | | |
| 1. Little interest or pleasure in doing things | | □None | | □Several days | | | □Over half of the time | | | □Almost everyday | |
| 2. Feeling down, depressed, or hopeless | | □None | | □Several days | | | □Over half of the time | | | □Almost everyday | |
| 3.Trouble falling asleep, staying asleep, or sleeping too much | | □None | | □Several days | | | □Over half of the time | | | □Almost everyday | |
| 4. Feeling tired or having little energy | | □None | | □Several days | | | □Over half of the time | | | □Almost everyday | |
| 5. Poor appetite or overeating | | □None | | □Several days | | | □Over half of the time | | | □Almost everyday | |
| 6. Feeling bad about yourself, or- that you’re a failure or have let yourself or your family down | | □None | | □Several days | | | □Over half of the time | | | □Almost everyday | |
| 7. Trouble concentrating on things, such as reading the newspaper or watching television | | □None | | □Several days | | | □Over half of the time | | | □Almost everyday | |
| 8. Moving or speaking so slowly that other people could have noticed. Or, the opposite - being so fidgety or restless that you have been moving around a lot more than usual | | □None | | □Several days | | | □Over half of the time | | | □Almost everyday | |
| 9. Thoughts that you would be better off dead or of hurting yourself in some way | | □None | | □Several days | | | □Over half of the time | | | □Almost everyday | |
| **3.9a Have you been depressed and lost interest for two weeks?**  □No  □Yes **→**If you choose this option, Answer **3.9b**  **3.9b Have you been depressed and lost interest for two weeks during pregnant and postnatal period? （Answer, if you choose “1”、“2”、“3 and more” in 1.10c）**  □No □Yes | | | | | | | | | | | |
| **3.10 In the past 2 weeks, how often do you experience the following events? GAD-7 Anxiety** | | | | | | | | | | | |
| 1. Feeling nervous, anxious or on edge | | □None | | □Several days | | | □Over half of the time | | | □Almost everyday | |
| 2. Not being able to stop or control worrying | | □None | | □Several days | | | □Over half of the time | | | □Almost everyday | |
| 3. Worrying too much about different things | | □None | | □Several days | | | □Over half of the time | | | □Almost everyday | |
| 4. Trouble relaxing | | □None | | □Several days | | | □Over half of the time | | | □Almost everyday | |
| 5. Being so restless that it is hard to sit still | | □None | | □Several days | | | □Over half of the time | | | □Almost everyday | |
| 6. Becoming easily annoyed or irritable | | □None | | □Several days | | | □Over half of the time | | | □Almost everyday | |
| 7. Feeling afraid as if something awful might happen | | □None | | □Several days | | | □Over half of the time | | | □Almost everyday | |
| **3.11 In the past 1 week, how often do you experience the following events? SCL-90 OCD** | | | | | | | | | | | |
| 1. Unwanted thoughts, etc., that won’t leave your mind. | | □Not at all | | | □Mild | | □Moderate | □Serious | | | □Extremely |
| 2. Worried about sloppiness or carelessness | | □Not at all | | | □Mild | | □Moderate | □Serious | | | □Extremely |
| 3. Having to do things very slowly to insure correctness. | | □Not at all | | | □Mild | | □Moderate | □Serious | | | □Extremely |
| 4. Having to check and double check what you do. | | □Not at all | | | □Mild | | □Moderate | □Serious | | | □Extremely |
| 5. Having to repeat the same actions, i.e., counting, washing. | | □Not at all | | | □Mild | | □Moderate | □Serious | | | □Extremely |
| **3.12 The following questions are about your sleeping *in the past 2 weeks*. Please answer the question according to your condition in the past month. Please choose options that match your situation best. ISI Insomnia** | | | | | | | | | | | |
| **1. How long do you usually sleep at night in the past 2 weeks?**  □≤5h □6h □7h □8h □9h □≥10h | | | | | | | | | | | |
| **2. The severity of your current (last 2 weeks) insomnia problem:：** | | | | | | | | | | | |
| a. Difficulty falling asleep | □Never □Sometimes □Always | | | | | | | | | | |
| b. Difficulty staying asleep | | □Never | | | | □Sometimes | | | □Always | | |
| c. Problems waking up too early | | □Never | | | | □Sometimes | | | □Always | | |
| **3. Are you satisfied with your sleeping in the past 2 weeks？**  □Very satisfied □Relatively satisfied □Fair □Dissatisfied □Very dissatisfied | | | | | | | | | | | |
| **4.** **Do you have a habit of napping at lunch breaks?**  □No □Yes **→**If you choose this option, Answer **4a**  **4a. How long do you nap at lunch break**  □ <30min □30~60min □61~90min □＞90min | | | | | | | | | | | |
| **5.In the past 2 weeks, how often do you use sleeping medication?**  □No  □＜1 time/week **→**If you choose this option, Answer **5a**  □1-2 times/week **→**If you choose this option, Answer **5a**  □ ≥3 times/week **→**If you choose this option, Answer **5a**  **5a. Source of your sleeping medication:**  □After consultation or seeing a doctor with prescription □Medicate by self | | | | | | | | | | | |
| **3.13** **Have you been diagnosed with the following conditions?** | | | | | | | | | | | |
| Depression | | | □No □Yes | | | | | | | | |
| Anxiety | | | □No □Yes | | | | | | | | |
| Obsessive-compulsive disorder | | | □No □Yes | | | | | | | | |
| Other psychiatric disorder | | | （Please specify ） | | | | | | | | |
| **3.14 Do you have family history of psychiatric and mental disorders (Including parents, grandparents, siblings)**  □No □Yes **→**If you choose this option, continue to choose：□Parents □Grandparents □Siblings □ Prefer not to answer | | | | | | | | | | | |
| **3.15 Have you went a psychiatric or psychiatric clinic?**  □No **→**If you choose this option, Answer **3.15a**  □Yes  □ Prefer not to answer  **3.15a If you have a psychological problem, will you seek professional help?**  □Yes □Uncertain □No | | | | | | | | | | | |
| **3.16 Is there have a mental healthcare organization in the hospital you are working at?**  □No □Yes □Unknow | | | | | | | | | | | |
| **3.17 *In the past year,* have you participated in mental health promotion related activities?**  □No □Yes | | | | | | | | | | | |
| **3.18 Have you been diagnosed the following diseases？** | | | | | | | | | | | |
| 1. High blood pressure □No □Yes | | | | | | | | | | | |
| 2. Diabetes □No □Yes | | | | | | | | | | | |
| 3. Hyperthyroidism □No □Yes | | | | | | | | | | | |
| 4. Hypothyroidism □No □Yes | | | | | | | | | | | |
| 5. Gastric/duodenal ulcer □No □Yes | | | | | | | | | | | |
| 6. Polycystic ovary syndrome (PCOS) □No □Yes | | | | | | | | | | | |
| 7. Autoimmune diseases (Hashimoto's thyroiditis, graves' disease, systemic lupus erythematosus, Sjogren's syndrome, compulsory spondylitis, etc.)  □No □Yes | | | | | | | | | | | |
| 8. Malignant tumors □No □Yes (Specify the diagnosis ) | | | | | | | | | | | |
| 9. Other diseases □No □Yes (Specify the diagnosis ) | | | | | | | | | | | |
| **3.19 *In the past year,* do you have persistent or recurring pain for more than 3 months?**  □No  □Yes**（If you choose this option, answer 3.19a）**  **3.19a Your main pain area is [multiple selection]**  □ Head  □ Face  □ Shoulder and neck  □ Lower back  □ Abdomen  □ Hip and knee joints  □ Pain all over the body  □ Other | | | | | | | | | | | |
| **3.20 *In the past year*, have you been hospitalized due to illness?** □No □Yes | | | | | | | | | | | |

| **Section 4. Lifestyle** |
| --- |
| **4.1 Smoke**  **4.1a *In the past year,* how often do you smoke?**  □ Never or occasionally (less than 1 cigarette per day)  □ Smoked before (smoked more than 1 cigarette per day, for more than a year, quitted)  □ Smoking (smoking more than 1 cigarette per day, for more than a year)  **4.1bHave you ever lived with a smoker for 6 months or more?**  □never  □I have, length of residence：______years (Please fill in integers）  □Currently living together for more than 6 months, length of residence：______years (Please fill in integers）  **4.1c** *I****n the past year,* your average number of days of passive smoking per week (at least 5 minutes at a time):**  □None  □＜1 day  □1~2 days **→** If you choose this option, Answer **4.1d**  □3~5 days **→** If you choose this option, Answer **4.1d**  □≥6 days **→** If you choose this option, Answer **4.1d**  **4.1d** ***In the past year*，you smoked passively for______ hours per week on average?（Please fill in integers）** |
| **4.2 *In the past year,* how often do you drink alcohol?**  □Never □Once a month or less □2~4 times a month □ 2~3 times a week □ 4 times or more a week |
| **4.3 *In the past year,* how often do you drink milk tea?**  □Never □less than 1cup per week □1~2 cups per week □3~6 cups per week □more than 7 cups per week |
| **4.4 *In the past year,* how often do you exercise?**  □Never □ Once a week or less □ 2~3 times a week □ 4~5 times a week □6~7 times a week |
| **4.5 *In the past year,* do you keep a pet?**  □No □Yes **→**If you choose this option, Answer **4.15a**  **4.5a How often do you interact with your pet (walk a dog, pet a cat, and chat)?**  □Never □Seldom □Sometimes □Often □Always |
| **4.6 Which hand do you usually use when writing and eating with chopsticks?**  □ Left hand □ Right hand □ Both |

| **Section 5. Adverse Childhood Experience, Life Events** | | | | | | | |
| --- | --- | --- | --- | --- | --- | --- | --- |
| **5.1 Do you feel happy about your childhood?**  □Very happy □Happy □Fair □Unhappy □Extremely unhappy | | | | | | | |
| **5.2 How often did the following events happen in your childhood (before 16 years old)？：** | | | | | | | |
| 1.I felt loved (R) (emotional neglect) | □Never | □Several times | □Sometimes | □Often | □Always | | □ Prefer not to answer |
| 2.There was someone to take me to the doctor when I needed it (R) (physical neglect) | □Never | □Several times | □Sometimes | □Often | □Always | | □ Prefer not to answer |
| 3.People in my family hit me so hard, it left me with bruises or marks (physical abuse) | □Never | □Several times | □Sometimes | □Often | □Always | | □ Prefer not to answer |
| 4.I felt that somebody in my family hated me (emotional abuse) | □Never | □Several times | □Sometimes | □Often | □Always | | □ Prefer not to answer |
| 5.Somebody molested me (sexual abuse) | □Never | □Several times | □Sometimes | □Often | □Always | | □ Prefer not to answer |
| **5.3** ***In the past year,*** **have you had any of the following life events?**  ***LES-Life Events Scale*** | | | | | | | |
| 1. Overwhelmed (in terms of working/studying/living) | | | | □No | | □Yes | |
| 2. Death of a spouse/parent/relative | | | | □No | | □Yes | |
| 3. Family and marital problems (including quarrelling/fighting/separating/ breaking up with partner) | | | | □No | | □Yes | |
| 4. Relationship problems (troubles with boss/colleagues/neighbours) | | | | □No | | □Yes | |
| 5. Troubles in financial state (including liabilities, losses, theft) | | | | □No | | □Yes | |
| 6. Career/academic setbacks | | | | □No | | □Yes | |
| 7. Violations of the law | | | | □No | | □Yes | |
| 8. Troubles with the future of son or daughter (e.g., marriage, attending college, employment etc.) | | | | □No | | □Yes | |
| 9. Troubles with living conditions (including crowd, noise, relocation) | | | | □No | | □Yes | |
| 10. Outstanding personal achievement (including award, promotion, marriages, in-love) | | | | □No | | □Yes | |
| 11. Major injury or illness of a family member (including acute /chronic disease, severe hurt) | | | | □No | | □Yes | |
| 12. Major change in personal health or behaviour (including disease, hurt, surgery, sexual difficulties) | | | | □No | | □Yes | |
| 13. Other events not mentioned above | | | | □No | | □Yes (Specify the diagnosis ) | |

| **Section 6. Family and social relations** |
| --- |
| **6.1 How many intimate relatives do you have?**  □None □1~2 □3~5 □6~9 □≥10 |
| **6.2 How many close friends do you have?**  □None □1~2 □3~5 □6~9 □≥10 |
| **6.3 Do you have someone with whom you can share your joys and sorrows?**  □No  □Yes **→**If you choose this option, Answer **6.3a**  **6.3a How often do you meet or get in touch with her/him/them?**  □Everyday □Every week □Every month □Several times every year □Once a year or less |
| **6.4 When you have problems or make a difficult decision, can you get emotional support from others?**  □Always □Often □Sometimes □Seldom □Never |
| **6.5 In the past year, you live：**  □ Alone □With families □With friends or colleagues  □ Changing accommodations frequently, and most with strangers |
| **6.6 Have you ever received support and encouragement from your managers?**  □Always □Often □Sometimes □Several times □Seldom |
| **6.7 Have you ever received support and encouragement from your colleagues?**  □Always □Often □Sometimes □Several times □Seldom |
| **6.8 Have you ever received support and encouragement from your family?**  □Always □Often □Sometimes □Several times □Seldom |
| **6.9 In the past year, did you have a partner? (1.10 answered "Unmarried", "Divorced" and "Widowed"）**  □No □Yes **→**If you choose this option, Answer **6.9a** |
| **6.9a How often did you suffer violence from your partner in the past year?**  1. physical violence  □No □1 time □2~3 times □＞3 times □ Prefer not to answer  2. emotional violence  □No □1 time □2~3 times □＞3 times □ Prefer not to answer  3. sexual violence  □No □1 time □2~3 times □＞3 times □ Prefer not to answer |

| **Section 7. Current Affairs Opinions** | |
| --- | --- |
| **7.1 Did you donate blood?**  □No **→**If you choose this option, Answer **7.1a** □Yes  **7.1a Are you willing to donate blood？** □Yes □No | |
| **7.2 Are you willing to donate organs？** | □Yes □No |
| **7.3 Are you willing to provide part-time home care service?** | □Yes □No |
| **7.4 Are you in favor of nurse prescription rights？** | □Yes □No |
| **7.5 Are you in favor of euthanasia?** | □Yes □No |

| **Section 8. Overall Evaluation** | | | | | | |
| --- | --- | --- | --- | --- | --- | --- |
| **8.1 Following questions are about your life satisfaction *in the past year*, please tick the box** **that fits your situation. E.g., “☑”** | | | | | | |
| **1. Are you satisfied with your work？** | □Very satisfied | □Relatively satisfied | □Some satisfied | □Some not satisfied | □Relatively not satisfied | □Extremely not satisfied |
| **2. Are you satisfied with your finance？** | □Very satisfied | □Relatively satisfied | □Some satisfied | □Some not satisfied | □Relatively not satisfied | □Extremely not satisfied |
| **3. Are you satisfied with your family？** | □Very satisfied | □Relatively satisfied | □Some satisfied | □Some not satisfied | □Relatively not satisfied | □Extremely not satisfied |
| **4. Are you satisfied with your friendship？** | □Very satisfied | □Relatively satisfied | □Some satisfied | □Some not satisfied | □Relatively not satisfied | □Extremely not satisfied |
| **5. Are you satisfied with your health？** | □Very satisfied | □Relatively satisfied | □Some satisfied | □Some not satisfied | □Relatively not satisfied | □Extremely not satisfied |
| **8.2 In general, do you feel happy about your life？**  □Very happy □Happy □Fair □Not happy □Extremely not happy | | | | | | |

Thank you for your patience!

The information you provide will be a valuable contribution to the promotion of nurses' health!

Please provide your contact information. After the questionnaire is submitted, you will receive a copy of the information provided by us, and you will also have the opportunity to win 100 yuan cash-filled red envelopes!

Once again, we sincerely appreciate your participation and dedication. Your support is the greatest driving force for our progress!

Tel：**□□□□□□□□□□□**

The last four digits of the ID number：**□□□□**

E-mail：

Your suggestions on nurses' mental health:
